# Weak and Maneuvering Target Detection with Long Observation Time Based on Segment Fusion for Narrowband Radar

**DOI:** 10.3390/s22187086

**Published:** 2022-09-19

**Authors:** Shaopeng Wei, Yan Dai, Qiang Zhang

**Affiliations:** 1 College of Oceanography and Space Informatics, China University of Petroleum (East China), Qingdao 257099, China; 2 Beijing Institute of Radio Measurement, Beijing 100854, China; 3 Beijing Institute of Tracking Telecommunications Technology, Beijing 100094, China

**Keywords:** long CPI coherent integration, target detection, TBD, updated CFAR

## Abstract

Detecting high-speed and maneuvering targets is challenging in early warning radar applications. Modern early warning radar has many functions such as detection, tracking, imaging, and recognition which need a high signal-to-noise ratio (SNR). Thus, long-time coherent integration is a necessary method to realize high SNR requirements. However, high-speed and maneuverable motion cause range and Doppler migration, which brings about serious coherent integration loss. Traditional integration methods usually have the drawbacks of model mismatching and high computational complexity. This paper establishes a novel long coherent processing interval (CPI) integration algorithm that detects maneuvering and weak targets which have a low reflection cross-section (RCS) and low echo SNR. The range and Doppler migration problems are solved via a layer integration by blending the association in a tracking-before-detection (TBD) technique. Compact SNR gain is achieved with a target information transmission mechanism and an updated constant false alarm ratio (CFAR) threshold. The algorithm is applicable in multiple target scenarios by considering different velocity ambiguities and maneuvers. A simulation and real-measured experiments confirm the effectiveness of the algorithm.

## 1. Introduction

With the development of aerospace technology, the detection of space targets has attracted much attention in recent years. Small space targets, including missile shrapnel and precision-guided weapon seekers, pose a considerable threat to defense security. If these targets are detected, it will significantly reduce threats to our safety. Thus, detecting small space targets greatly challenges our early warning radar. Space targets are usually characterized by a low reflection cross-section (RCS), which results in a low SNR. Therefore, coherent or incoherent integration in a long CPI is an effective way to obtain a high SNR gain. However, space targets usually have high-speed and maneuverable motion, leading to range and Doppler migration and causing severe performance loss of integration gain [1,2,3]. Estimating motion parameters and compensating for the motion phase is an effective way to obtain fully coherent integration gain and detect space targets successfully.

In recent years, many methods have been proposed to realize coherent or incoherent integration of high-speed and maneuverable targets. Generally, they can be divided into three categories: (1) Incoherent integration method. A Hough transform (HT) [4,5,6] is a typical incoherent integration method, which finds the slot of the envelope exceeding the detection threshold and integrates the signal along tracks to realize long time incoherent integration. However, the non-coherent SNR gain loss leads to performance degradation in very low SNR conditions. (2) Decoupling of range frequency and slow time. After down-conversion and pulse compression, the targets’ motion related to slow time couples with range frequency in the range frequency-slow time domain. As for uniform motions, only the linear coupling phase needs to be considered. The typical keystone transformation method [7,8,9] modifies the slow time to decouple the range frequency and slow time for high-speed targets. After decoupling, the range frequency and slow time are independent, and thus, the signal can be coherently integrated into the fast time and Doppler domains independently. For maneuverable target detection, many modified keystone transformation methods have been developed, such as the high-order keystone transformation [10,11,12] and deramp-keystone transformation [13,14], which aim to be a specific motion model to realize decoupling and coherent integration. Another decoupling method is based on Radon transformation [15,16,17], which jointly searches range and velocity parameters to realize decoupling. These methods can estimate high-order motion model parameters such as acceleration and jerk [18,19]. (3) Time-frequency analysis method. Unlike methods (1) and (2) that integrate in the fast time and Doppler domain, this method uses specific time-frequency distribution to realize parameter estimation and energy accumulation. Lv’s distribution (LVD) [20,21,22,23] uses a scaled correlation between the adjacent slow times. This way, the quadratic phase can be converted to a linear phase, and the echo can be integrated into the frequency-chirp rate domain. The method breaks through the tradeoff between resolution and cross-term [24], and parameter searching is unnecessary. The method has good performance in the low SNR scene. The Wigner–Ville distribution (WVD) [25,26,27] is a typical time-frequency analysis method to detect constant acceleration motion. With the changing of slow time, the acceleration produces a chirp rate modulation, and the velocity has a centroid frequency related to slow time in the echo phase. In this case, the echo can be treated as a linear frequency modulation signal (LFM) in slow time. Therefore, the time-frequency analysis method points to this feature to integrate echo in the time-frequency domain. By using the WVD time-frequency analysis method, the target echo can be well integrated. The modified WVD method has been studied in [28,29,30]. Though the time-frequency analysis method can estimate and accumulate low SNR target signals, some limitations should be noticed. The time-frequency transform is non-orthogonal, and the side lobes cannot be ignored. In conclusion, these traditional methods share problems of model limitations and high computational complexity.

Except for signal domain processing, recently, the tracking-before-detection (TBD) methods [4,31,32,33,34,35,36] have been studied, which combine target detection with tracking. Unlike traditional tracking-after-detection methods that use target estimation parameters to track, TBD methods use original signal data. Combined with the signal and target motion transition formulation, the energy can be integrated along the target’s motion tracks. After integration, the tracks can be outputted when the integration amplitude exceeds the CFAR threshold. There are four categories of TBD methods: dynamic programming (DP), the recursive Bayesian approach, the finite-set statistics theory (FISST) method, and the histogram-probabilistic multi-hypothesis tracker (H-PMHT). However, the TBD method usually uses incoherent integration along the target tracks, and thus, the technique loses performance when the single pulse is a low SNR condition.

Based on the weak target detection consideration, there are two critical problems: model mismatching and high computational complexity. The problem of model mismatch arises because the target cannot keep the same motion model in a long CPI, and it needs a higher-order model to fit the motion model. Given these problems, we propose a robust and efficient layer integration algorithm to detect weak and maneuvering radar targets with a long CPI for narrowband radar. This paper uses the piecewise way to simplify the complex motion over a long time. First, the echoes over a long time are divided into several echo segments over a short time. The complex motion over a long time can be regarded as combining several different uniform motions in a short time. Then, the coherent integration for every signal segment is carried out, and a low detection threshold is used to obtain the rough detection results in every signal segment. Because of every segment’s short coherent integration time, many false alarms are in the detection results. A target association and signal integration between segments using the H-PMHT are proposed to increase SNR gain and exclude false alarms. The high-order motion parameter is estimated based on the velocity variation between target association results. Because the target can be associated and the target’s signal can be integrated between segments, and as the false alarms are hardly integrated, the false alarms can be excluded. The target can be detected with the segment fusion layer by layer. In addition, to avoid the target glint problem, the target information transmission policy is also designed to prevent the loss of target information. Compared with traditional methods, the proposed method mainly has three advantages:

(1) It uses piecewise integration and TBD between slow-time segments to increase model adaption and decrease computational complexity;

(2) The layer integration detection mode, updated CFAR threshold, and target-alarm transmission association mechanism are designed so that the glint targets can be detected effectively;

(3) It uses coherent integration processing and compensates for the motion parameters which are estimated by associating between segments, helping to detect the low SNR targets effectively.

The rest of paper is organized as follows: In Section 2, the algorithm framework is built, and in Section 3, the method is introduced extensively, including the signal model, threshold detection, target association and target-alarm information base design. In Section 4, the algorithm’s complexity and limitations are discussed. In Section 5, some simulation experiments are designed. Finally, some conclusions are summarized in Section 6.

## 2. Algorithm Framework

**Figure 1:**
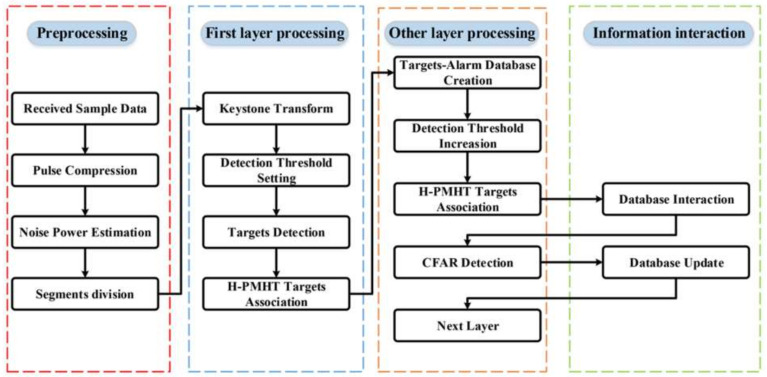
Overall processing flow chart.

**Figure 2:**
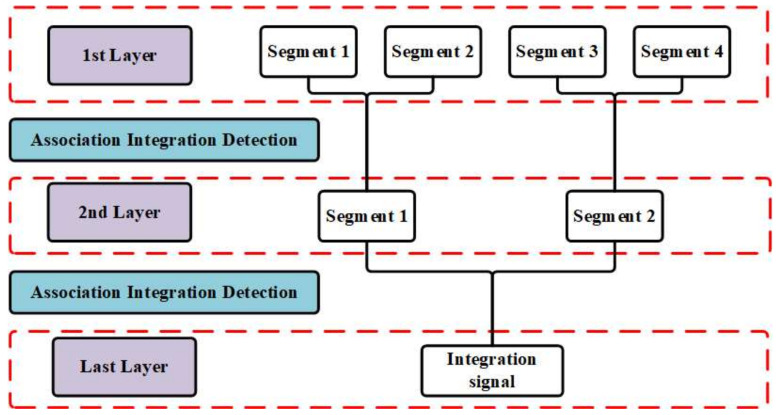
Layer integration mode.

Figure 1 shows the flow chart of the method of layered target detection based on information transmission and the updated threshold, and Figure 2 shows the layer integration mode. The algorithm can be summarized as the segment division that decreases the order of motion model to realize the coherent integration in short periods, which offers enough of an SNR to ensure the correctness of the TBD association. Furthermore, since the low detection threshold results in many false alarms, updating the increased threshold excludes the non-integration alarms. Moreover, the target-alarm transmission machine ensures that the glint target information can be transmitted with each layer increasing. The process is divided into five steps as follows:

(1). Preprocessing: The pulse compression and segment division are based on radar parameters in slow time. To detect all targets, we must select a certain number of pulses to obtain enough of an SNR and ensure that the target cannot have Doppler migration in every segment. The specific method of segment determination is discussed in the following sections.

(2). Noise power estimation and low-threshold detection: Range migration is corrected using the keystone transform in every slow-time segment. The noise power is estimated, and the CFAR threshold is set to detect targets based on noise power. To make sure that all targets can be detected, the threshold should be low, which means that many false alarms may be involved; thus, the increased threshold with layers is necessary to exclude false alarms and preserve the target.

(3). Target association and interaction: Combined with the TBD method, targets can be associated between segments. The target usually has the case of glint RCS, which means that the targets cannot be detected in a specific segment, and the target information cannot be transmitted into the following layer. The problem can be solved by designing a target-alarm information transmission mechanism to preserve targets and alarm information in every layer so that the information on the glint target would not be discarded as the layers increase.

(4). Compensation and integration: Compensate for the range and Doppler phase of the associated targets using the estimated parameters and coherently integrate them. Then, the integrated segments are transmitted to the next layer and processed using the same steps.

(5). CFAR threshold updating: With the increasing layers, the targets are integrated, and the alarms are not. Thus, the CFAR threshold needs to be increased based on the integration gain.

The algorithm was summarized above, and the specific algorithm is described in the next section.

## 3. Layer Integration and Detection Method with Information Transmission and Threshold Updating Algorithm Description

In Section 2, we described our overall algorithm framework. In this section, we discuss the algorithm specifically using mathematical derivation from four aspects, which are the layer signal model, integration and detection, tracking and association, and the information transmission mechanism.

### 3.1. Layer Signal Model

Suppose that radar transmits an LFM signal:

(1) $$s(t)=\mathrm{rect}\left(\frac{t}{T_{p}}\right)\exp\left[j\left(2\pi f_{c}t+\pi \gamma t^{2}\right)\right]$$

where rect[⋅] is the rectangle window function, $T_{P}$ is the pulse width, $f_{c}$ is the radar’s carrier frequency, and γ is the chirp rate. There are K targets with a high-order motion in the scenario, and the k-th target motion formulation can be expressed as:

(2) $$R_{k}(\tau )=R_{0k}-v_{k}\tau -a_{k}\tau^{2}-h_{k}\tau^{3}\cdots ,$$

where τ is the slow time. The received signal of targets can be written as follows after down-conversion:

(3) $$s_{r}(t,\tau )=\sum_{k=1}^{K}\sigma_{k}\mathrm{rect}\left(\frac{t-R_{k}(\tau )}{T_{p}}\right)\cdot \exp\left[-j\frac{4\pi f_{c}R_{k}(\tau )}{c}\right]\cdot \exp\left[j\pi \gamma {\left(t-\frac{2R_{k}(\tau )}{c}\right)}^{2}\right].$$

The echo in the range frequency-slow time domain (f,τ) can be written as:

(4) $$s_{r,c}(f,\tau )=P(f)\sum_{k=1}^{K}\sigma_{k}\mathrm{rect}\left(\frac{f}{B}\right)\cdot \exp\left[-j\frac{4\pi (f+f_{c})R_{k}(\tau )}{c}\right]$$

where B is the signal’s bandwidth, and P(f) is the Fourier transform of the LFM signal. By multiplying Equation (4) by $P{\left(f\right)}^{*}$ (* denotes conjugate), the signal model is given by [14]:

(5) $$s_{r,c}(f,\tau )={\left|P(f)\right|}^{2}\sum_{k=1}^{K}\sigma_{k}\mathrm{rect}\left(\frac{f}{B}\right)\cdot \exp\left[-j\frac{4\pi (f+f_{c})R_{k}(\tau )}{c}\right].$$

From Equation (5), we can see that the phase is nonlinear due to maneuverable motion. We use the piecewise concept to adapt to high-order complex motion models, which use several uniform motions in continuous slow time to approximate high-order motions. This way, we divide the long CPI into several segments, thus the phase can be approximately regarded as linear and integrated using the keystone transform. Moreover, the assumption of the piecewise segmental method is reasonable since narrowband radar usually has a low pulse repetition frequency (PRF) to reduce range ambiguity. Combined with dividing several segments of slow time, every segment’s velocity resolution is low. Thus, it can be regarded as a constant velocity with a low-velocity resolution. Moreover, the acceleration error can be compensated for by using the estimation parameter of the segment association. We use the long CPI to integrate signals to get a high SNR gain. However, it will bring substantial computational costs if we process the signal during the slow time. For example, suppose the keystone transform is used to correct the range migration. In that case, the computational complexity is proportional to the square of the slow-time number using the discrete Fourier transform (DFT) method. Therefore, if we use the long CPI to obtain coherent integration results, the computational burden usually is unacceptable. On the contrary, the segment division decreases the complexity affected by the number of pulses, and parallel computing can be used in every segment to increase calculation efficiency. Then, we derive the signal model based on the segment division.

Suppose that there are N pulses in one CPI, and the signal transmits to the l-th layer and has $n_{l}$ segments. In this case, the number of pulses in the l layer is $N_{l}=\frac{N}{n_{l}}$, and the q-th segment’s slow time can be represented as:

(6) $$\tau (l,q,p)=(q-1)N_{l}T_{r}+pT_{r},p=1,2,3\ldots N_{l},$$

where $T_{r}$ is the pulse repetition period. Based on the piecewise consideration, the segment division should ensure that the acceleration motion cannot cause the Doppler migration in every segment. In this way, the additional phase caused by acceleration should be less than π/4. For the practical detection scene, the maximum acceleration has a fixed bound. For example, the acceleration of flight is less than 10 ${m/s}^{2}$. Therefore, we make the target’s maximum acceleration value $a_{\max}$, and the maximum phase caused by the acceleration can be represented as:

(7) $$angle\left\{\exp\left(-j\frac{4\pi }{c}f_{c}\left(\frac{1}{2}a_{\max}{\left(\frac{N}{2}\Delta T\right)}^{2}\right)\right)\right\}<\frac{\pi }{4}$$

where $angle\left\{\cdot \right\}$ is the calculator to calculate the angle of the complex value and N is the pulse number in one segment. Thus, it can be equivalent to:

(8) $$\begin{array}{l} \frac{1}{2}a_{\max}{\left(\frac{N}{2}\Delta T\right)}^{2}<\frac{\lambda }{16}\Rightarrow \\ N<\sqrt{\frac{\lambda }{2a_{\max}\Delta T^{2}}} \end{array}$$

Further, in order to ensure the maximum energy integration, the pulse number of one segment can be determined by:

(9) $$N=\left\lfloor \sqrt{\frac{\lambda }{2a_{\max}\Delta T^{2}}}\right\rfloor$$

where $\left\lfloor \cdot \right\rfloor$ is rounded down to an integer. Substituting Equation (6) into Equation (5), we have:

(10) $$s_{r,c}\left(f,l,q,p\right)={\left|P(f)\right|}^{2}\sum_{k=1}^{K}\sigma_{k}\mathrm{rect}\left(\frac{f}{B}\right)\cdot \exp\left\{-j\frac{4\pi \left(f+f_{c}\right)R_{k}\left[\tau \left(l,p,q\right)\right]}{c}\right\}.$$

Suppose that the radar maximum non-ambiguous velocity is $v_{\max}$, the number of ambiguity is $n_{v}$, and the velocity without ambiguity is $v_{0}$. Then, the motion equation of each segment can be written as:

(11) $$v_{k}(l,q)=n_{v}v_{\max}+v_{0k}+a_{k}(q-1)N_{l}T_{r}.$$

(12) $${R^{\prime }}_{k}\left(l,q,p\right)=R_{0k}\left(l,q\right)-v_{k}\left(l,q\right)pT_{r}.$$

Thus, the signal model in the q-th segment of the l-th layer can be written as:

(13) $$s_{r,c}(f,l,p,q)={\left|P(f)\right|}^{2}\sum_{k=1}^{K}\sigma_{k}\mathrm{rect}\left(\frac{f}{B}\right)\cdot \exp\left\{-j\frac{4\pi (f+f_{c}){R^{\prime }}_{k}\left[\tau (l,p,q)\right]}{c}\right\}.$$

In this way, we use the segment division method to realize model matching and reduce the computational burden problem. Thus, the signal can be decoupled and integrated in every segment using the keystone transformation as subsection B.

### 3.2. Keystone Uncoupling and Updating CFAR Threshold

After segmentation division and piecewise approximation using the method in subsection A, the nonlinear phase can be regarded as a linear phase coupled with the range frequency in a short time period. The keystone transformation modifies the slow time axis to decouple the range frequency and slow time from the coupling phase; this specific method references [11]. Note that the keystone transformation is performed without the ambiguous velocity; however, the velocity of the target is usually ambiguous in low frequency radar, thus the ambiguous velocity should be compensated in the different ambiguity channels. The number of ambiguity can be determined by the energy of the ambiguous channel output.

The ambiguous channel of the keystone signal model unfolding Equation (10) is:

(14) $$\begin{array}{l} s_{r,c}\left(f,l,p,q\right)={\left|P(f)\right|}^{2}\sum_{k=1}^{K}\sigma_{k}\mathrm{rect}\left(\frac{f}{B}\right)\cdot \\ \;\;\;\exp\left\{-j\frac{4\pi (f+f_{c})\left[R_{0k,l,q}-\left(n_{v}v_{\max}+v_{0k}\left(l,q\right)\right)pT_{r}\right]}{\lambda }\right\}, \end{array}$$

where $v_{0k}\left(l,q\right)$ is the velocity without ambiguity. The compensation function of the ambiguous velocity can be expressed as:

(15) $$H^{c}(f,n_{v})=\exp\left[j\frac{4\pi }{\lambda }(1+\frac{f}{f_{c}})(n_{v}v_{\max}pT_{r})\right].$$

The signal after the ambiguity compensation is:

(16) $$\begin{array}{l} s^{\left(1\right)}{}_{r,c}(f,l,p,q){=s}_{r,c}(f,l,p,q)\cdot H^{c}(f,n_{v})\\ =\sum_{k=1}^{K}\sigma_{k}\mathrm{rect}\left(\frac{f}{B}\right)\cdot \exp\left\{-j\frac{4\pi (f+f_{c})\left[R_{0k,l,q}-v_{0k}(l,q)pT_{r}\right]}{c}\right\}. \end{array}$$

By applying the keystone transformation, and substituting $(f+f_{c})pT_{r}=f_{c}p{T^{\prime }}_{r}$ into Equation (13), the signal can be written as:

(17) $$\begin{array}{l} s^{\left(1\right)}{}_{r,c}\left(f,l,p,q\right)={\left|P(f)\right|}^{2}\sum_{k=1}^{K}\sigma_{k}\mathrm{rect}\left(\frac{f}{B}\right)\\ \cdot \exp\left[-j\frac{4\pi (f+f_{c})R_{0k,l,q}}{c}\right]\cdot \exp\left[j\frac{4\pi v_{0k}(l,q)pT_{r}{}^{\prime }}{\lambda }\right] \end{array}$$

The keystone transformation can be processed by the chirp Z-transform-inverse Fourier transform (CZT-IFFT) or scaled Fourier transform-inverse Fourier transform (SFT-IFFT) method to improve operational efficiency. Therefore, the signal can be integrated by moving the target detection (MTD) after the keystone transformation.

After integrating the first layer, the CFAR detection threshold should be determined based on noise power. The high speed and maneuvering target detection are usually applied in detecting space targets such as missiles and aircraft. The space target detection usually has these characteristics: (1) the clutter has little effect on echo; (2) the echo is mainly affected by the thermal noise of the transmitter and receiver; (3) the distribution of noise with different ranges and Doppler cells is usually homogeneous. In this case, the influence of clutter on the detection is small, and only Gaussian white noise is considered. Assuming that all the measurement cells share the same noise distribution, based on the CFAR detection theory, the adaptive threshold corresponding with the constant false alarm rate and statistical noise power should be applied to the detector. As for this condition, the white Gaussian noise obeys $n(t)\sim N(0,\sigma^{2})$, and the amplitude obeys the Rayleigh distribution after envelope detection. The possibility density function is:

(18) $$f\left(A|H_{0}\right)=\frac{A}{\sigma^{2}}\exp\left(\frac{-A^{2}}{2\sigma^{2}}\right),$$

where $H_{0}$ is the hypothesis that there are no targets in the detection cell and A is the amplitude of echo. Based on the Neyman–Pearson criterion, the alarm rate can be deduced as [37]:

(19) $$P_{fa}=\int_{V_{T}}^{\infty }\frac{A}{\sigma^{2}}\exp\left(\frac{-A^{2}}{2\sigma^{2}}\right)dA=\exp\left(\frac{-V_{T}^{2}}{2\sigma^{2}}\right),$$

where $V_{T}$ is the detection threshold. Thus, the detection threshold can be represented as $V_{T}=\sqrt{2\sigma^{2}\ln\left(\frac{1}{P_{fa}}\right)}$. As for the noise power estimation, we can choose the range cells in the far distance to estimate it, since the echo power is proportional to $1/r^{4}$, where r is the range between the cell and radar. The power of the target in the far distance is much lower than the noise and can be ignored. The noise power can be estimated by these cells as:

(20) $${\hat{\sigma }}^{2}=\frac{1}{W}\sum_{w=1}^{W}{\left|s_{w}\right|}^{2},$$

where W is the number of reference cells needed in order to estimate noise. Thus, the threshold can be determined by noise power estimation. It is noted that a low threshold should be used in the first layer to ensure that all the targets can be detected. However, the low threshold means a high false alarm rate and many false alarms involved. Updating the increased threshold is necessary to exclude false alarms. Assume that the threshold in the l−th layer is $V_{T}^{l}$, and the number of pulses in one segment of this layer is $N_{l}$. The target can obtain a $10\log_{10}\left(N_{l}{}^{2}\right)$ dB gain of power by coherent integration and the noise obtains a $10\log_{10}\left(N_{l}\right)$ dB gain of power. Based on the difference in the integration gain, the threshold power can be increased by $10\log_{10}\left(N_{l}{}^{2}\right)$ dB to distinguish the target and false alarm. Therefore, the threshold in l+1−th can be determined by:

(21) $$V_{T}^{l+1}=10^{\left[10\log_{10}\left({\left(V_{T}^{l}\right)}^{2}\right)+10\log_{10}\left(N_{l}^{2}\right)\right]}.$$

In this way, with the layer increasing, the targets can exceed the increased threshold and false alarms cannot since the false alarms have a low probability of being associated with the layer detector passing by; therefore, the false alarms will be excluded and the target will be integrated and detected in the last layer.

### 3.3. H-PMHT Association and Integration

After segment division, integration, and target detection in every segment, the signals need to be integrated between segments. The targets have a high-speed and maneuverable motion, resulting in the range and velocity change in the adjacent segment. Based on the detection and integration result, the problem becomes associating targets between segments, compensating for the phase caused by the maneuverable motion, and integrating them. Combined with the TBD concept, we use the H-PMHT method to associate targets. The H-PMHT method quantizes the amplitude of measurement cells as a synthetic histogram with several shots. The shot is treated as a measurement, and the sum of shots is treated as the total number of acquiring measurements. The shot distribution of a segment obeys multinomial distribution, where the probability of each cell can be considered as a superposition of targets and noise. Moreover, successive scans are associated with the state transformation function. Thus, the joint probability distribution can be obtained, and parameters can be estimated using the expectation maximization (EM) method. Moreover, [37,38] derived that the Kalman filter can be used to estimate parameters, including motion parameters and amplitude, in the subsequent scans, whereas [39,40] revealed that the estimated intensity can indicate whether or not the association is successful. The target will be treated as an alarm if the estimated intensity drops below 0 dB.

Suppose that targets have constant acceleration between segments; then, the state transformation function in the l-th layer can be expressed as:

(22) $$x_{p+1}=\left[\begin{array}{ccc} 1 & N_{l}T_{r} & \frac{{\left(N_{l}T_{r}\right)}^{2}}{2}\\ 0 & 1 & N_{l}T_{r}\\ 0 & 0 & 1 \end{array}\right]x_{p}+\varepsilon ,$$

where the p-th and p+1-th target states in the segment are $x_{p}={\left[r_{p},v_{p},a_{p}\right]}^{T},x_{p+1}={\left[r_{p+1},v_{p+1},a_{p+1}\right]}^{T}$, and $\left\{r,v,a\right\}$, which represent the range, velocity, and acceleration observation value, respectively. ϵ is the state error. The acceleration can be denoted by $a_{p}=a_{p+1}=\frac{v_{p+1}-v_{p}}{N_{l}T_{r}}$. The measurement function can be stated as:

(23) $$y_{p+1}=\left[\begin{array}{ccc} 1 & 0 & 0\\ 0 & 1 & 0 \end{array}\right]x_{p+1}.$$

Based on the H-PMHT, the distribution of the mean cell value is given by [40]:

(24) $$P(r,v)=\sum_{k=0}^{K}\pi_{k}P_{k}(r,v),$$

where $P_{k}(r,v)$ is the state distribution of the k−th target, and when k=0, it is noise distribution. We assume that the noise distribution is uniform in the detection cells, thus the pdf of noise is:

(25) $$P_{0}=\frac{1}{N_{R}\cdot N_{V}},$$

where $N_{R}$ and $N_{V}$ are the number of range cells and number of Doppler cells. As for target distribution, it is reasonable that we assume the range and velocity distribution obeys the normal density function with the variance $\sigma_{r}$ and $\sigma_{v}$. Thus, the k-th target contribution can be expressed as [39]:

(26) $$P_{k}(r,v)=N(R_{k},\sigma_{r})N(V_{k},\sigma_{v}),k=1,2\ldots K.$$

The state and proportion estimations can be updated by using recursive implementation. By combining the intensity and state estimations, we can confirm whether or not the targets are associated, and the specific algorithm is summarized as Algorithm [40].

Targets have been associated between segments (q,q+1) using the TBD method. The next step is to compensate for the range and velocity migration, and to integrate the signal between segments. Suppose that the target state in two segments can be represented as $\left(r_{1},v_{1}\right)$ and $\left(r_{2},v_{2}\right)$, the signal in two segments can be derived as follows:

(27) $$\begin{array}{l} s_{r_{1},v_{1}}=\sigma_{k}\mathrm{rect}\left(\frac{f}{B}\right)\cdot \exp\left[-j\frac{4\pi \left(f+f_{c}\right)r_{1}}{c}\right]\cdot \exp\left[j\frac{4\pi v_{1}(qn_{l}{T^{\prime }}_{r}+pT_{r}{}^{\prime })}{\lambda }\right]\\ s_{r_{2},v_{2}}=\sigma_{k}\mathrm{rect}\left(\frac{f}{B}\right)\cdot \exp\left[-j\frac{4\pi (f+f_{c})r_{2}}{c}\right]\cdot \exp\left[j\frac{4\pi v_{2}\left((q+1)n_{l}{T^{\prime }}_{r}+pT_{r}{}^{\prime }\right)}{\lambda }\right]. \end{array}$$

Thus, the compensation function can be written as:

(28) $$\begin{array}{l} H^{r}=\exp\left[-j\frac{4\pi (f+f_{c})(r_{1}-r_{2})}{c}\right]\\ H^{v}=\exp\left[j\frac{4\pi (v_{1}-v_{2})\left((q+1)n_{l}{T^{\prime }}_{r}+pT_{r}{}^{\prime }\right)}{\lambda }\right]\\ H^{a}=\exp\left[j\frac{4\pi \left(a{\left(\left(q+1\right)n_{l}T_{r}{}^{\prime }+pT_{r}{}^{\prime }\right)}^{2}\right)}{\lambda }\right]. \end{array}$$

After compensating for the motion phase of targets based on Equation (23), the signal can be coherently integrated.

### 3.4. Target Transmission Mechanism

**Figure 3:**
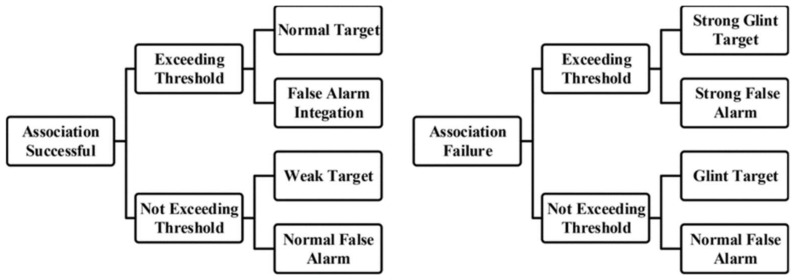
Cases of target association and detection.

The signal can be integrated using the above method in long coherent periods; however, there is an additional question to consider regarding glint target integration and detection. The glint target usually has a low RCS in a specific segment, and the echo cannot be integrated effectively. Based on the layer detection framework, the false alarms which cannot exceed the increased threshold will be discarded and result in missing the detection of the glint target, which is not integrated and treated as a false alarm. Considering this question, if we preserve the false alarm information and design the mechanism of information transmission and interaction, the glint targets could be detected. From the target detection analysis, Figure 3 shows all the possible situations after association and integration.

According to the above analysis, false alarms are unlikely to be associated between segments. Even though they can be successfully associated with a specific layer, they are not likely to pass by the final layer and exceed the threshold. Therefore, only the cases of the targets’ association and detection should be considered to avoid missing detection. The association and detection of targets can be divided into four issues as follows:

(1) Targets are well associated and exceed the threshold (normal targets). It is an ideal condition, and the target can be detected in the last layer;

(2) Targets are well associated and do not exceed the threshold (weak targets). The target is unstable and the integration gain is less than the increased threshold, thus the target’s information should be preserved and transmitted into the following layers;

(3) Targets are not associated and exceed the threshold (strong glint targets). Although the target is glint, the power is strong enough in a specific segment, and the target also can be detected in the following layers;

(4) Targets are not associated and do not exceed the threshold (glint targets). If the target is glint and weak, the target will not be associated and exceeds the threshold. Thus, it cannot be detected in the following layer, and the information should be preserved.

**Figure 4:**
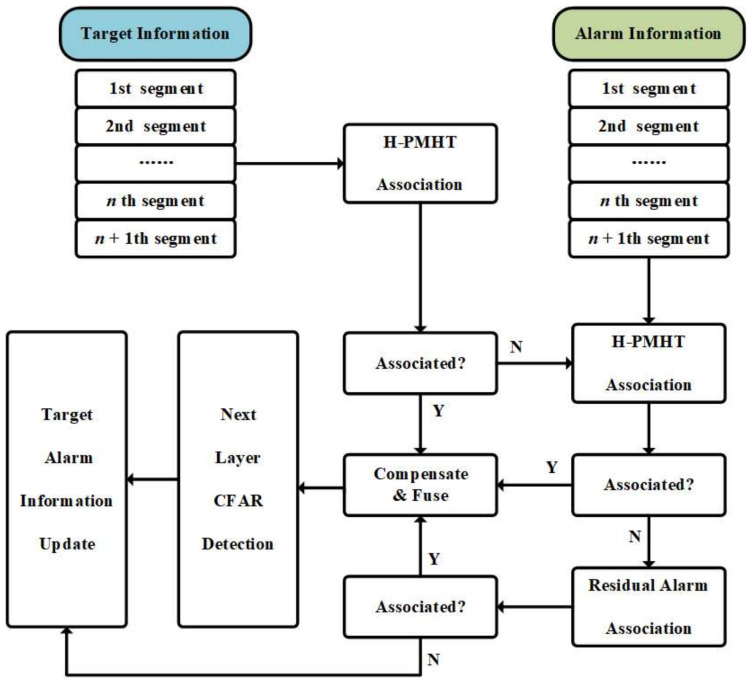
The transmission and interaction mechanism based on detection result. (Y is yes, and N is no).

As for these four types of targets, the target and false alarm pursuit mechanism is designed as shown in Figure 4. In short, the alarm information will be preserved, and the association steps between targets and false alarms will be added to complete the association of type (2) and (4) targets. In a nutshell, the association method is designed with three stages: (a) associate between detected targets, which ensures that normal targets can be associated; (b) non-associated targets associate with false alarms, which makes sure that weak or glint targets can be integrated; and (c) associate between false alarms, which makes sure that the glint target which is not integrated and detected can be associated. This way, the four kinds of targets are associated and transmitted into the final layer to be detected. The specific processing method refers to Figure 4.

## 4. Algorithm Complexity and Parameter Determination

### 4.1. Computational Complexity

As for computational complexity, the LVD method is an effective way to solve the maneuvering motion integration problem. It needs a correlation process and keystone transformation in every range cell. Suppose that $N_{r}$ is the number of range cells, $N_{v}$ is the number of velocity cells, and M is the number of ambiguous channels. The computational complexity is $O\left(N_{r}MN_{v}{}^{2}\log\left(N_{v}\right)\right)$ [20]. The proposed method needs to implement one keystone transformation in the first layer. Moreover, it does not add the signal dimension, and the number of targets and alarms determines the complexity of the association. Suppose the number of targets is K, the number of segments is $n_{l}$, the number of false alarms has an expectation $\frac{N_{v}N_{r}}{n_{l}}P_{fa}$ and $N_{a}$ is the number of iterations; thus, the proposed method’s computational complexity is less than:

(29) $$O\left\{M\left[N_{r}N_{v}\log\left(N_{v}\right)+\left(K+\frac{N_{r}N_{v}}{n_{l}}P_{fa}\right)N_{a}\log_{2}\left(n_{l}\right)\right]\right\}.$$

Therefore, our method is more efficient compared with the LVD distribution method. Compared with parameter searching methods such as the Radon Fourier transform [16], our approach only needs to search ambiguous parameters. Thus, many repetition complements are omitted to increase calculation efficiency. Based on the complexity analysis, the radar parameters are set as follows: carrier frequency—1 GHz; bandwidth—15 MHz; pulse repetition frequency—2000 kHz; the number of pulses—960; the number of range cells—100; and false alarm rate—$10^{-7}$. In this case, we set a single target scenario and used an Intel^®^ Core^TM^ i7-7700 CPU and Matlab 2017 to compare the proposed method with the LVD method for calculation efficiency. The LVD method uses 31.779885 s on average to calculate one range cell. However, the total calculation time of the proposed method is 5.344031 s. The results show that the proposed method is more efficient than the correlation method, such as LVD.

### 4.2. Parameter Determination

A. The threshold of the first layer determination. The first layer threshold should be determined by the lowest signal power in a single pulse. Assume that the lowest SNR of a target after pulse compression to be detected is $P_{0\min}$ dB, and the number of integration pulses in the first layer is $N_{1}$. In this case, the target’s power after coherent integration of the first layer is $P_{0\min}+10\log_{10}\left(N_{1}\right)$. If the target with minimum power can be detected in the first layer detector, the threshold $V_{T}^{1}$ should meet the requirement as:

(30) $$V_{T}^{1}\leq 10^{\left[P_{0\min}+10\log_{10}\left(N_{1}\right)\right]\;}.$$

B. The number of pulses in the first layer segment determination. The number of pulses in the first layer is determined by two aspects: Doppler migration and the SNR requirement in the first layer detector. The Doppler migration limitation makes sure that the target echo satisfies the piecewise assumption. The SNR requirement makes sure that the target which has the lowest power requirement can be detected. Based on these two criterions, the number of pulses can be determined as follows:

(1) Doppler migration limitation. Based on the uniform motion assumption, the target cannot cross the Doppler cell in the segment. The Doppler migration is caused by an equivalent acceleration, and the maximum equivalent acceleration is defined as $a_{\max}$ in the realistic application. Assuming that the number of pulses is $N_{1}$ in the first layer, the pulse repetition frequency is $f_{r}$, and the wave length is λ, the target with the maximum acceleration should satisfy the condition:

(31) $$\frac{a_{\max}N_{1}}{f_{r}}\leq \frac{f_{r}\lambda }{2N_{1}}$$

Simplify it, and the limitation can be derived as:

(32) $$N_{1}\leq \sqrt{\frac{\lambda f_{r}{}^{2}}{2a_{\max}}}.$$

(2) The SNR requirement in the first layer detection. The SNR can be increased by $10\log_{10}\left(N_{1}\right)$ dB after $N_{1}$ coherent integration pulses in the first layer. Assuming that the lowest SNR of a target that can be detected in the first layer is $Q_{1\min}$ dB, then the minimum SNR of the single pulse $Q_{0\min}$ should meet the requirement:

(33) $$Q_{0\min}\geq Q_{1\min}-10\log_{10}\left(N_{1}\right).$$

Therefore, the $N_{1}$ can be determined as:

(34) $$N_{1}\geq 10^{\left(Q_{1\min}-Q_{0\min}\right)/10}.$$

The number of pulses $N_{1}$ in the first layer segment can be determined by the above two limitations. However, there is a situation where the $N_{1}$ based on the SNR criterion is bigger than the Doppler criterion. In this case, we should increase the motion order in the segment, such that we assume that the target has constant acceleration in every segment and has constant jerk between segments. In this way, the number of pulses $N_{1}$ can be increased to make sure that it satisfies the Doppler and SNR criterions at the same time.

## 5. Experiment Analysis

The experiment comprises three parts to prove that our method is effective for multiple targets, especially glint target integration and detection. First, a scenario of multiple targets with different range velocity accelerations and ambiguity numbers is set to prove that our method is valid. Second, a glint target scenario is set to verify that our approach has better performance for glint target integration compared with the traditional method. An integration comparison experiment uses our technique and traditional techniques to analyze integration and detection performance. Third, Monte Carlo experiments are designed to quantitatively compare the detection probability and integration SNR.

### 5.1. Integration SNR Upper Limit

In order to make a reference to integration performance, first we derive the upper limit of the pulse compression and coherent integration signal. Suppose that the length of transmitting sampling is $L_{s}$, and the RCS of the target is $A_{t}$; thus, the signal’s power $P_{s}$ after pulse compression is:

(35) $$P_{s}=L_{s}A_{t}{}^{2}.$$

We assume that the noise power spectral density is $P_{n}$ and the SNR after pulse compression can be derived.

(36) $$SNR_{PC}=\frac{P_{s}}{P_{n}}=10\log_{10}\left(\frac{L_{s}A_{t}{}^{2}}{P_{n}}\right).$$

If we coherently integrate the N pulse compression signal, the ideal integration SNR can be written as:

(37) $$SNR_{out}^{ideal}=SNR_{PC}+10\log_{10}(N)$$

Whatever integration method we use, the integration SNR output cannot exceed Equation (34); thus, we can use the SNR upper limit to evaluate the integration method performance.

### 5.2. Case of Multiple Target Integration and Detection

**Figure 5:**
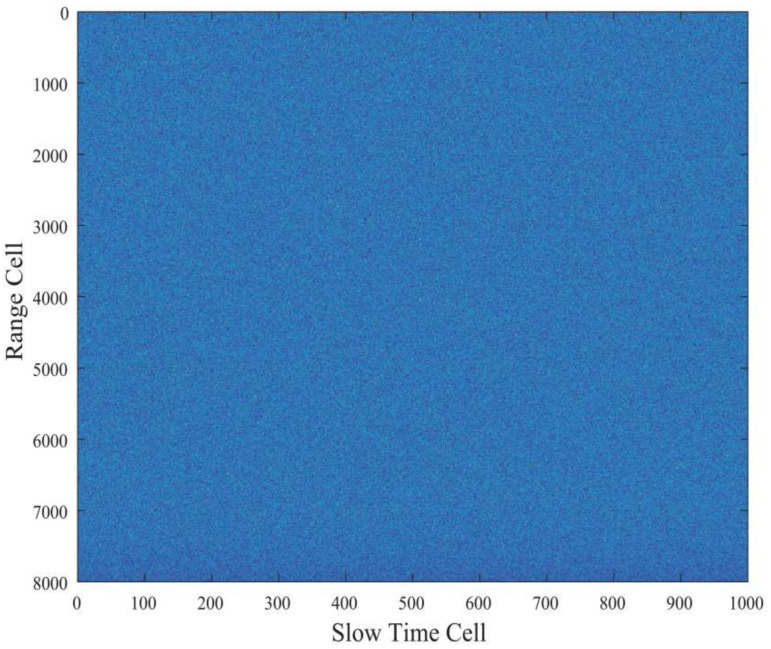
The pulse compression result.

To prove that our algorithm is effective in the aspects of multi-targets, multi-ambiguity channels, glint targets, and low SNR detection, the scenario that includes three targets is set to evaluate the multi-target detection performance. The ranges of target1, target2, and target3 are in the 2000th, 1000th, and 300th cells, and the velocities are 600 m/s, 1600 m/s, and 2300 m/s, respectively. The accelerates of the targets are 60 m/s^2^, 60 m/s^2^ and 20 m/s^2^, respectively. Based on the radar parameters shown in Table 1, target1 is in the zeroth ambiguity channel, and target2 and target3 are in the first ambiguity channel. In this way, we can verify the ability of the multi-ambiguity channel processing. Third, target1 is the glint target, whose RCS is too low to be detected in the segments. As for the target glint, it is assumed that the glint segment obeys binomial distribution. The glint probability is set as 0.25. The glint target integration and detection ability can be tested in this case. Finally, we set the single-pulse SNR to be −33 dB to verify the low SNR detection performance. Using the above target scenario to test the integration and detection performance, this experiment can test the performance of multi-targets, multi-ambiguity channels, glint targets, and low SNR conditions. Figure 5 shows the results of echo pulse compression. Before the coherent integration, the tracks of targets cannot be seen in Figure 5 under the low SNR condition.

**Figure 6:**
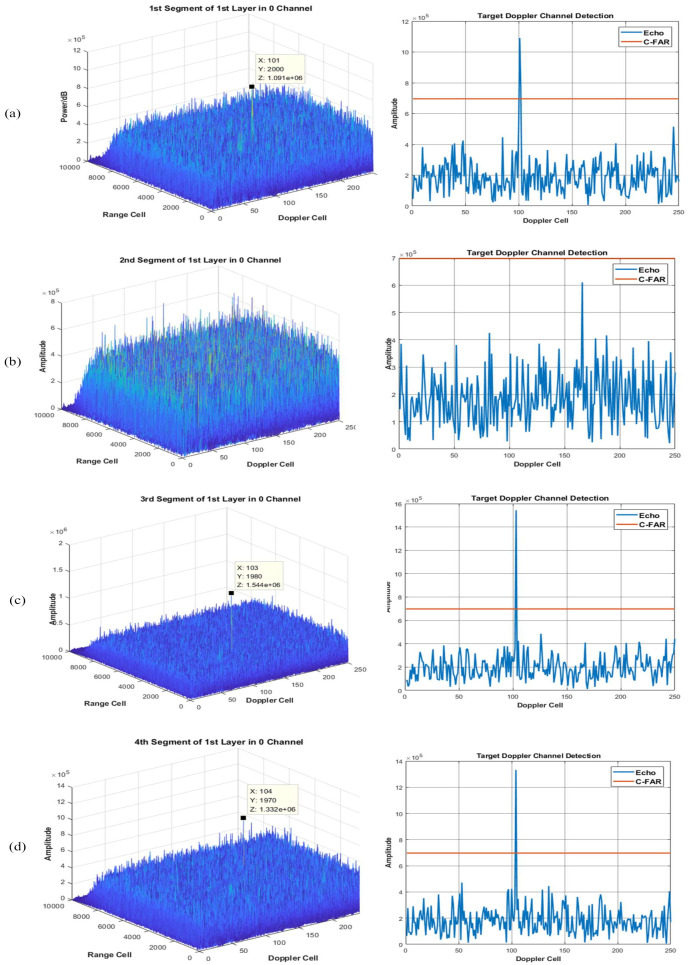
The 0th ambiguity channel and the 1st layer output result. (**a**) is the result of integration and detection in the 1st segment; (**b**) is the result of integration and detection in the 2nd segment; (**c**) is the result of integration and detection in the 3rd segment; (**d**) is the result of integration and detection in the 4th segment.

**Figure 7:**
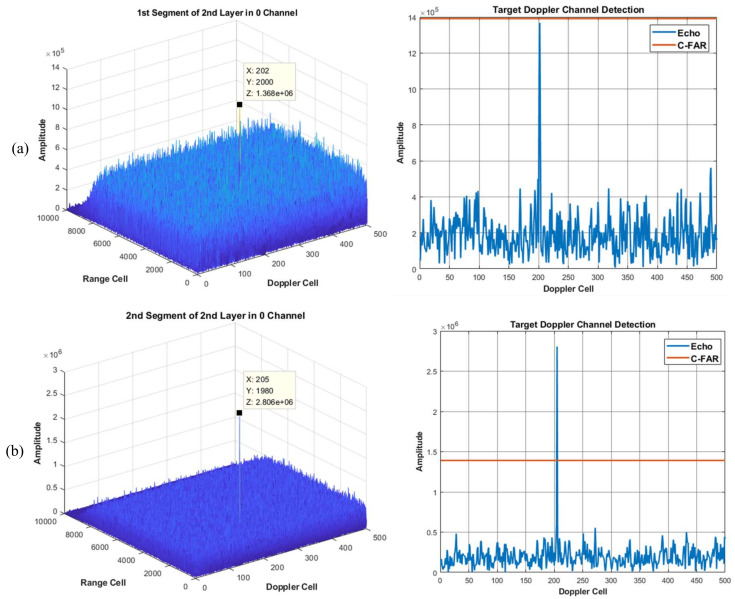
The 0th ambiguity channel and the 2nd layer output result. (**a**) is the result of integration and detection in the 1st segment; (**b**) is the result of integration and detection in the 2nd segment.

**Figure 8:**
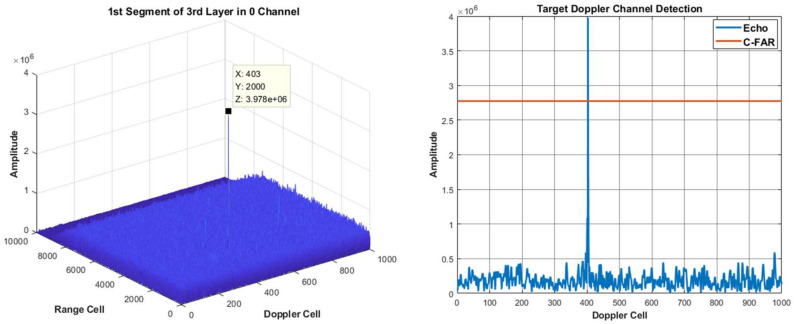
The 0th ambiguity channel and the 3rd layer integration and detection result.

**Figure 9:**
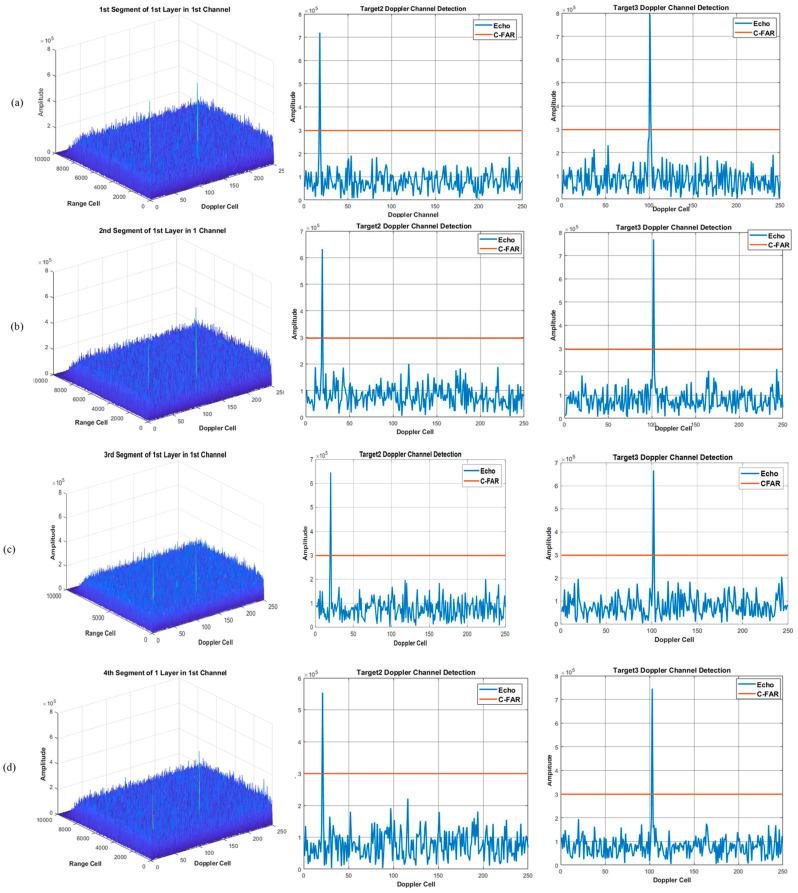
The 1st ambiguity channel and the 1st layer output result. (**a**) is the result of integration and detection in the 1st segment; (**b**) is the result of integration and detection in the 2nd segment; (**c**) is the result of integration and detection in the 3rd segment; (**d**) is the result of integration and detection in the 4th segment.

**Figure 10:**
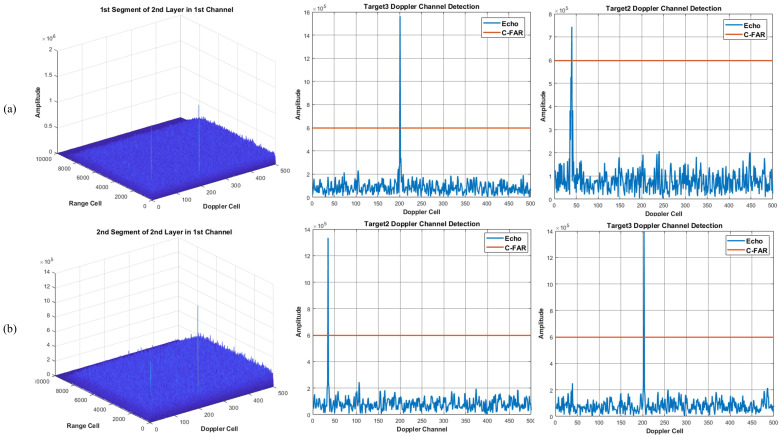
The 1st ambiguity channel and the 2nd layer output result. (**a**) is the result of integration and detection in the 1st segment; (**b**) is the result of integration and detection in the 2nd segment.

**Figure 11:**
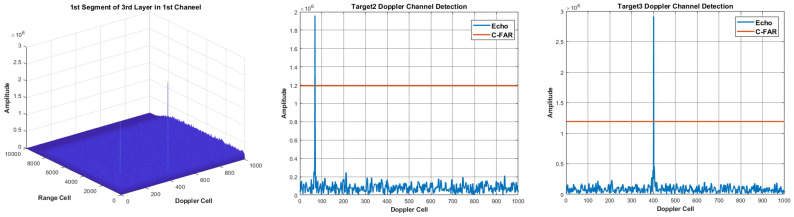
The 1st ambiguity channel and the 3rd layer integration and detection result.

Using the proposed method of integration and detection to obtain the visualized integration and detection result in every layer, a three layer detector is set. In other words, the original signal can be divided into four segments. Figure 6, Figure 7 and Figure 8 show the integration and detection results of every layer in the zeroth ambiguity channel, whereas Figure 9, Figure 10 and Figure 11 show the integration and detection results of every layer in the first ambiguity channel. From Figure 6, only target1 energy can be effectively integrated after using an ambiguity match filter. Moreover, due to the target glint, the target1 cannot be combined and detected in the second segment of the first layer, as shown in Figure 6b, but the target1 in the rest of the segments of the first layer can be detected, as shown in Figure 6a,c,d. Therefore, the target cannot be associated with the first and second segments in the first layer. From Figure 7a, as the threshold increases, the target cannot be detected in the first segment of the second layer. Thus, the target is treated as a false alarm. Although target1 can be detected in the second segment of the second layer, the information of target1 in the first segment of the second layer will be lost. However, due to the design of the target and alarm information transmission mechanism, the information of target1 can be transmitted successfully. The target1 in the first segment can be associated with the second layer detector. After parameter estimation and compensation, the target1 is integrated with the last layer and exceeds the previous detection threshold to be detected successfully, as shown in Figure 8. If the transmission mechanism is not designed, the information of target1 will be discarded in the second layer, which results in missing detection. As for the integration and detection result in the first ambiguity channel, from Figure 9, Figure 10 and Figure 11, target2 and target3 without glint are in the same ambiguity channel. After ambiguity matching, they can be well integrated and detected in the first layer, as shown in Figure 9. The motion formulation can be determined to realize the association based on the detection result. The two targets are well associated due to precise parameter estimation and are transmitted to the following layers, as shown in Figure 10. In this case, the targets without glint can be integrated and detected in the last layer from Figure 11. In conclusion, based on the experiment results, the ambiguity channel matching filter solves the problem of high-speed ambiguity. The piecewise segment division realizes integration in a short period, and the association between segments ensures that motion parameters can be determined and the acceleration motion can be compensated for. By transmitting with layers, the targets can be well integrated. Moreover, the information transmission and perseveration mechanism can ensure that the glint target is associated with the segment in all layers. Due to one experiment having a high random error, the quantitative integration and detection performance is not discussed in this subsection. The specific integration performance of glint and non-glint targets will be analyzed in Section 3 and Section 4.

### 5.3. Case of Glint Target Integration Performance Analysis

**Figure 12:**
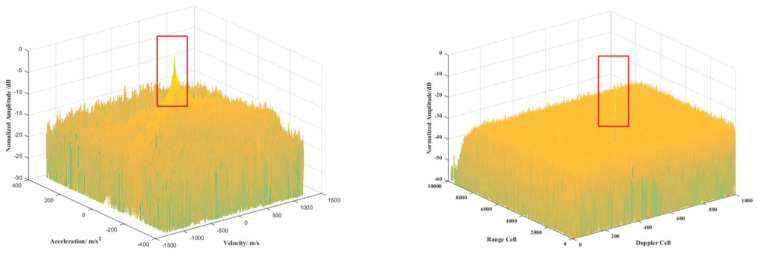
The result of glint target integration; the left figure is proposed method, the right figure is LVD method.

The target-alarm information transmission mechanism is designed as above. In this subsection, we design a comparison experiment with the LVD distribution method to prove that the proposed method has good integration performance with the glint target. Suppose that the target has a weak RCS in a specific segment, the glint case obeys binomial distribution with a probability of 0.25, and the radar parameters are set as shown in Table 1. The target is in the 2000th range cell. The velocity is 450 m/s, and the acceleration is 50 m/s^2^. A random simulation shows the target glint in the 251st–500th slow-time period. The proposed method and LVD distribution method integration results are shown in Figure 12 and Table 2.

From Figure 12, it can be seen that the glint target can be well integrated using the proposed method. The integration case with the LVD method can also realize coherent integration, but the integration SNR is much lower than the proposed method. Moreover, the LVD method has higher side lobes than the proposed method, resulting in the missing detection of a weak target in the side lobe area. As for the quantitative analysis shown in Table 2, the result shows that the integration SNR loss is 0.91 dB using the proposed method compared with LVD, which has a 11.8 dB SNR loss. This experiment proves that the proposed method performs better in glint target integration and detection. The reasons might be as follows: (1) The proposed method uses segment processing, and the signal is divided into several segments to be coherently integrated into every segment. It makes sure that the segment without glint can be sufficiently integrated, but LVD uses a correlation operation and decreases the SNR. (2) The proposed method uses an information transmission mechanism to be adapted to the target glint model, which makes sure that the target in every segment can be transmitted into all layers. (3) As for the LVD method, it uses a WVD and keystone transform to complete the correlation operation. The missing signal causes a significant effect on the WVD operation due to model mismatching. Based on the above reasons, the integration SNR has a considerable loss by using LVD, and the proposed method has a more robust ability for model matching.

### 5.4. Case of Quantitative Performance

**Figure 13:**
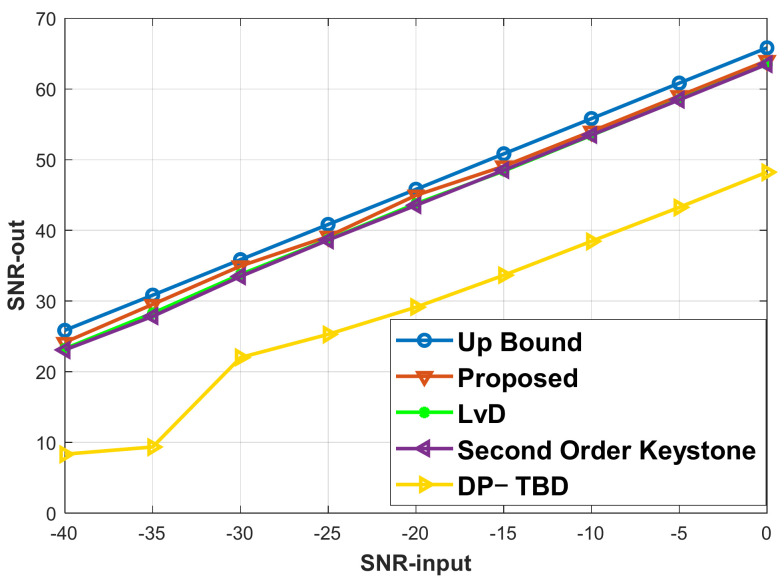
The output SNRs with different input SNRs.

In this subsection, we analyze a quantitative experiment’s integration and detection performance because the proposed method mainly solves the problem of SNR integration loss and the high complexity of the correlation method. In addition, the proposed method uses the TBD concept to realize association and integration. Therefore, it is necessary to compare the proposed method with traditional correlation and TBD detection methods. This subsection uses the traditional correlation method of LVD and the traditional TBD method of DP as comparative methods. The radar parameters are set as shown in Table 1. Suppose that the target velocity is 450 m/s, the acceleration is 50 m/s^2^, and 100 Monte Carlo trails are implemented. The results of output SNRs with different input SNRs are shown in Figure 13. The integration up bound can be easily determined using Equations (32)–(34), shown as the blue line in the figure. The SNRs of a single pulse before the pulse compression are set from −40 to 0 dB to test the output signal SNR using different methods. From the experiment results, the proposed method has the highest SNR output compared with other methods. The SNR integration loss is less than 1 dB compared with the upper limit. The LVD method has an integration SNR loss of about 3 dB compared with the integration up bound. The DP method has the worst performance because it uses the incoherent integration method. Our method has a better performance compared with other methods due to the reasons as follows: The LVD method uses the correlation between slow times, and the noise correlation causes the SNR loss. Moreover, the cross-terms in the correlation calculation bring about a high side lobe. In this case, the output SNR gain has a performance loss. On the other hand, the LVD method ignores the range migration caused by acceleration. Thus, it will cause model mismatching and result in SNR loss; DP uses the motion formulation to search the estimation area based on transmission states, and it uses incoherent integration to explore and determine the targets’ tracks when the final integration result is compared with the detection threshold. In conclusion, the estimation precision of motion parameters and incoherent integration limits the performance of the DP method. Therefore, the incoherent integration gain is much lower than a coherent integration method such as LVD and the proposed method, as the proposed method uses a piecewise concept to minimize model error. Moreover, the association and integration between segments solve the shortage of SNR loss, which comes from correlation. On the other hand, the proposed method combines TBD tracking with coherent integration and solves the problem of low SNR gain using the traditional DP method.

### 5.5. Real-Measured Data Analysis

The real-measured data of the space target are used to verify that the proposed method effectively completes signal integration for weak targets. The radar parameters are listed in Table 3.

**Figure 14:**
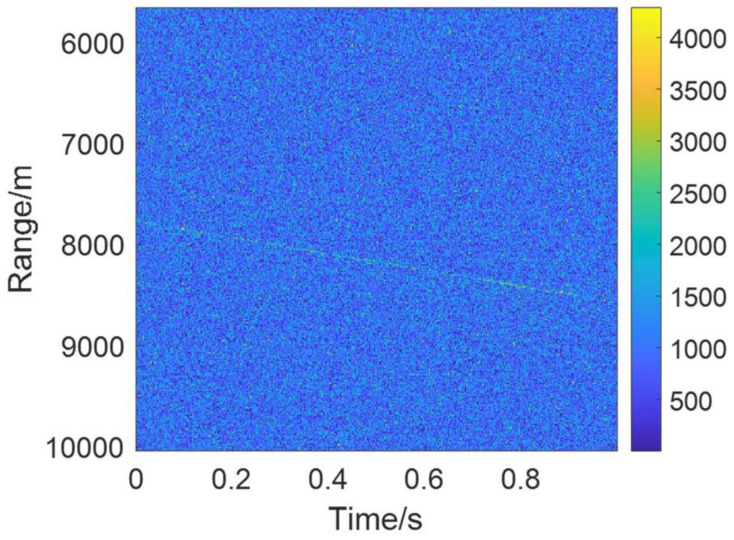
Real-measured data for space target.

The recorded echoes are shown in Figure 14. The original echoes have a very low SNR and we almost cannot find the target’s tracks in Figure 14.

**Figure 15:**
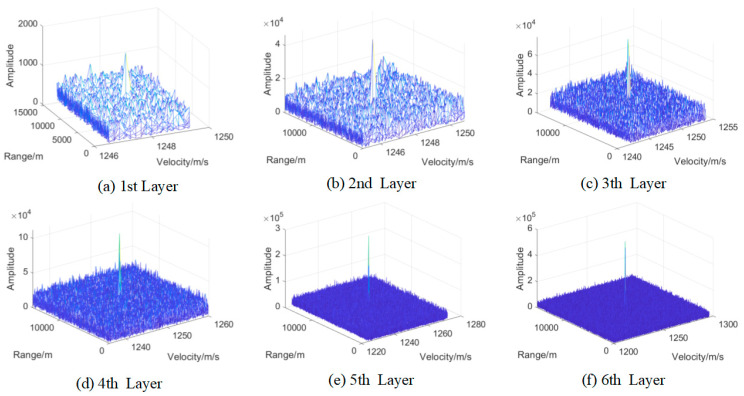
The integration results of every layer. (**a**–**f**) is the integration result of 1 to 6 layer, respectively.

For this target, we use the proposed detection method of segment division to realize coherent integration. The detection false alarm rate in the first layer is set as $10^{-2}$ and the false alarm rate in the last layer is set as $10^{-6}$, and we output the integration results of every layer, which is shown in Figure 15.

**Figure 16:**
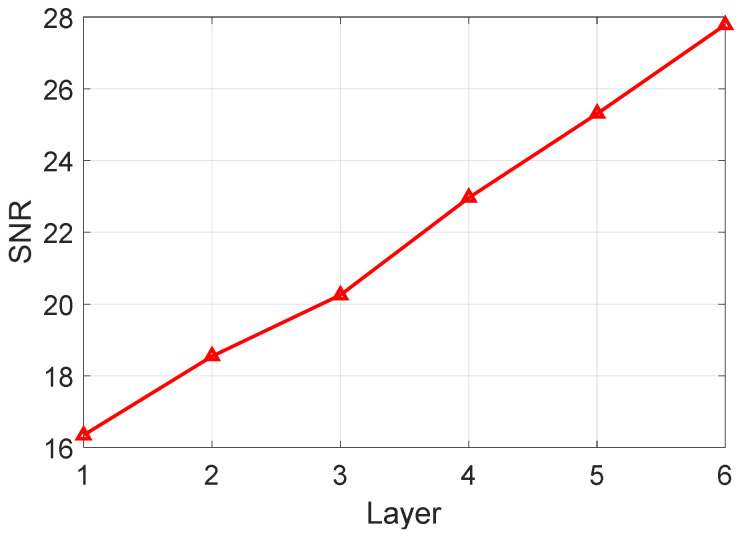
Integration SNR of every layer. The red line is the integration SNR of every layer.

From the results, we can see that the energy of the target is integrated layer by layer, and the false alarm cannot be integrated and excluded. In order to verify that the target is integrated gradually in every layer, the statistical SNR is calculated in Figure 16. From the results, we can see that the proposed method successfully realizes target integration and detection for the space target in real-measured data.

Finally, the estimation results of range, velocity, and acceleration are also given in Table 4. In all, based on the real-measured results, the proposed method can be used in practical applications.

## 6. Conclusions

Using the piecewise approximation mindset and segment integration method, the proposed method has advantages in increasing SNR gain and computational efficiency for small and weak targets. As seen in the simulated and real-measured experiments, the proposed method has more than 1 dB of SNR integration gain compared with traditional methods such as LVD. In addition, the computational speed increases almost 6 times more than in LVD. However, the proposed method relies on the robust detection results in the first layer, and a more complex motion will lead to the performance degradation of the proposed method. In addition, the target association is not considered good; thus, we will focus on robust initial detection and target association in future works.

## Figures and Tables

**Table 1:** Radar parameters in experiments.

Radar Parameters	Value
Carrier frequency	1 GHz
Bandwidth	5 MHz
Pulse width	75 μs
Pulse repetition frequency	500 Hz

**Table 2:** Integration SNR of glint target using LVD and proposed method.

Method	SNR Input	SNR Output
Ideal	−30 dB	34.63 dB
Proposed	−30 dB	33.72 dB
LVD	−30 dB	22.88 dB

**Table 3:** The real-measured radar parameters.

Radar Parameters	Value
Waveband	1 GHz
Bandwidth	1 MHz
PRF	800 Hz
Integration Time	1 s

**Table 4:** The results of target parameter estimation.

Range	Velocity	Acceleration
7770 m	1249.875 m/s	10.5469 m/s^2^

## Data Availability

Not applicable.
